# T cell function is dispensable for intracranial aneurysm formation and progression

**DOI:** 10.1371/journal.pone.0175421

**Published:** 2017-04-24

**Authors:** Haruka Miyata, Hirokazu Koseki, Katsumi Takizawa, Hidetoshi Kasuya, Kazuhiko Nozaki, Shuh Narumiya, Tomohiro Aoki

**Affiliations:** 1 Innovation Center for Immunoregulation Technologies and Therapeutics (AK project), Kyoto University Graduate School of Medicine, Kyoto, Japan; 2 Department of Neurosurgery, Shiga University of Medical Science, Shiga, Japan; 3 Core Research for Evolutional Science and Technology (CREST) from Japan Agency for Medical Research and Development (AMED), Kyoto University Graduate School of Medicine, Kyoto, Japan; 4 Department of Neurosurgery, Tokyo Women’s Medical University Medical Center East, Tokyo, Japan; 5 Department of Neurosurgery, Japan Red Cross Asahikawa Hospital, Hokkaido, Japan; Stellenbosch University Faculty of Medicine and Health Sciences, SOUTH AFRICA

## Abstract

Given the social importance of intracranial aneurysm as a major cause of a lethal subarachnoid hemorrhage, clarification of mechanisms underlying the pathogenesis of this disease is essential for improving poor prognosis once after rupture. Previous histopathological analyses of human aneurysm walls have revealed the presence of T cells in lesions suggesting involvement of this type of cell in the pathogenesis. However, it remains unclear whether T cell actively participates in intracranial aneurysm progression. To examine whether T cell is involved in aneurysm progression, intracranial aneurysm model of rat was used. In this model, aneurysm is induced by increase in hemodynamic force loaded on bifurcation site of intracranial arteries where aneurysms are developed. Deficiency in T cells and pharmacological inhibition of T cell function were applied to this model. CD3-positive T cells were present in human aneurysm walls, whose number was significantly larger compared with that in control arterial walls. Deficiency in T cells in rats and pharmacological inhibition of T cell function by oral administration of Cyclosporine A both failed to affect intracranial aneurysm progression, degenerative changes of arterial walls and macrophage infiltration in lesions. Although T cells are detectable in intracranial aneurysm walls, their function is dispensable for macrophage-mediated inflammation and degenerative changes in arterial walls, which presumably leads to intracranial aneurysm progression.

## Introduction

Subarachnoid hemorrhage attributable to rupture of intracranial aneurysms (IAs) has a poorest prognosis among all type of stroke in spite of recent diagnostic and therapeutic advances; a mortality rate of 50% and significant disabling effect in 50% of survivors [1, 2]. IA is a common pathology in intracranial arteries with a prevalence of 2–4% [3] in general public, and many IAs are indeed incidentally detected through MRI examination or some other modalities before rupture [4–6]..Nonetheless, currently, prevention of rupture of such incidentally found IAs is confined to surgical interventions like microsurgical clipping. However, because of the intrinsic risk of complication associated with surgical manipulations, interventions are performed only in less than half of cases with unruptured IAs [5]. Given the poor outcome due to subarachnoid hemorrhage after rupture of IAs and nature of unruptured IAs as an asymptomatic lesion, noninvasive prophylactic methods to treat IAs before rupture are socially demanded. Thereby, better understanding of the pathogenesis of IAs is essential. Recent accumulating evidence has established the notion that IA is a chronic inflammatory disease in intracranial arteries and macrophage plays a crucial role in the pathogenesis of IAs through regulating such inflammatory responses in lesions [7–11].

Histopathological studies of human IAs and IAs induced in experimental models have revealed the presence of T cells as second most abundant type of cells next to macrophage in lesions [12–14]. Further, Frosen et al. demonstrated that number of T cells infiltrated in walls of ruptured IAs is larger than those in unruptured IAs [15], indicating the association of T cells to rupture. Although T cell becomes a major constituent element that regulates immune and inflammatory responses in microenvironment of acquired immunity and thus contributes to the pathogenesis of variety of inflammatory or immune diseases, contribution of T cells to the pathogenesis of IAs and, if there is, how remains to be elucidated.

The aim of this study is to clarify whether T cell contributes to IA formation and progression by using rat model of IAs in combination with depletion of T cell or pharmacological inhibition of its function.

## Materials and methods

### Rodent IA models and histological analysis of induced IA

All of the following experiments, including animal care and use, complied with the National Institute of Health’s Guide for the Care and Use of Laboratory Animals and were approved by the Institutional Animal Care and Use Committee of Kyoto University Graduate School of Medicine.

Male F344/NJcl-rnu/rnu rats, which have a mutation in *Whn* gene resulting in absence of thymus and defect in T cells [16], and their wild type control, F344/Jcl rats, were purchased from Japan CLEA (Tokyo, Japan). Animals were maintained on a light/dark cycle of 14 h/10 h, and had a free access to chow and water. To induce IA, 7-week-old male rats were anesthetized by intraperitoneal injection of pentobarbital sodium (50 mg/kg) and subjected to ligation of the left carotid artery and systemic hypertension achieved by the combination of salt overloading and ligation of the left renal artery [17]. This surgical procedure is defined as the ‘aneurysm induction’. Immediately after the surgical manipulation, animals were fed a chow containing 8% sodium chloride. In a chow, 0.12% 3-aminopropionitrile (Tokyo Chemical Industry, Tokyo, Japan), an inhibitor of lysyl oxidase that catalyzes the cross-linking of collagen and elastin, was added to facilitate IA formation and progression [17]. Systemic blood pressure was measured by the tail-cuff method without any anesthesia and its value was calculated as a mean of independent 3 measurements per animal. At time points indicated in the corresponding Figure legends or Results after the aneurysm induction, animals were deeply anesthetized by intraperitoneal injection with a lethal dose of pentobarbital sodium, and transcardially perfused with the fixative, 4% paraformaldehyde. The right anterior cerebral artery (ACA) and olfactory artery (OA) bifurcation including the IA lesion was then stripped and serial frozen sections were prepared. IA induction, area of induced IAs and length between disrupted portion of internal elastic lamina at this bifurcation were histopathologically examined after Elastica van Gieson staining.

### Immunohistochemistry

Animals were sacrificed and serial frozen sections with 5 μm thickness were prepared as same. For immunohistochemistry, after blocking with 3% donkey serum (Jackson ImmunoResearch, Baltimore, MD), the sections were incubated with primary antibodies followed by incubation with secondary antibodies conjugated with fluorescent dye (Jackson ImmunoResearch). Finally, immunofluorescence images were acquired on a confocal fluorescence microscope system (Lsm-710, Carl Zeiss Microscopy GmBH, Gottingen, Germany).

Primary antibodies used are following; mouse monoclonal anti-smooth muscle alpha actin (α-SMA) antibody (#MS113, Thermo scientific, Waltham, MA), mouse monoclonal anti-CD68 antibody (#ab31630, Abcam, Cambridg, MA), rabbit polyclonal anti-matrix metalloproteinase (MMP) 9 antibody (#AB19016, Chmeicon, Temecula, CA), mouse monoclonal anti-CD3 antibody (#M7254, DAKO, Carpinteria, CA).

Macrophage was defined as a CD68-positive cell in immunohistochemistry. Thickness of media was defined as a ratio of the thickness of the thinnest portion in media, visualized by immunostaining for α-SMA, to that of the distal normal arterial wall.

### Cyclosporine A treatment

Cyclosporine A was purchased from Toronto Research Chemicals (#C988900, Ontario, Canada). Cyclosporine A was dissolved in olive oil to a final concentration and orally administered to rat IA models once a day. This dose of Cyclosporine A administered was determined by the preliminary experiment, in which Concanavalin A-induced increase in IL-2 concentration in plasma was suppressed by treatment with each dose of Cyclosporine A. Rats treated with Cyclosporine A were sacrificed at 14^th^ day after the aneurysm induction and sections of IAs induced at right ACA-OA bifurcation were prepared for further analyses.

### Primary culture of Bone marrow-derived macrophage (BMM)

Bone marrow cells were collected from femur of male F344/Jcl or F344/NJcl-rnu/rnu rats and cultured in Dulbecco’s Modified Eagle Medium (DMEM) supplemented with 20% fetal bovine serum (FBS) and macrophage-colony stimulating factor (M-CSF, 50 ng/ml, Peprotech, Rocky Hill, NJ) to differentiate into macrophages for 7 days. Cells were then harvested for further analysis.

### Quantitative real time (RT)-PCR analysis

RNA purification from primary culture of macrophages and reverse transcription of purified RNA were performed using a RNeasy Plus Mini Kit (QIAGEN, Hilden, Germany) and a High-capacity cDNA Reverse Transcription Kit (Life Technologies Corporation, Carlsbad, CA), according to manufacturers’ instructions. For the quantification of gene expression, quantitative RT-PCR was performed on a Real Time System CFX96 (Bio-rad, Hercules, CA) using a SYBR Premix Ex Taq II (TAKARA BIO INC., Shiga, Japan). β-actin was used as an internal control. For quantification, the second derivative maximum method was used for crossing point determination.

Primers used in the present experiment are listed in following; monocyte chemotactic protein-1 (MCP-1; *Ccl2*) forward 5’-TGCTGAAGTCCTTAGGGTTGATGC-3’ and reverse 5’-GCAGCAGGTGTCCCAAAGAAGC-3’; tumor necrosis factor-alpha (TNF-r; *Tnf*) forward 5’-CTCCTGGTATGAAGTGGCAAATCG-3’ and reverse 5’-TGGCATGGATCTCAAAGACAACC-3’; cyclooxygenase-2 (COX-2; *Ptgs2*) forward 5’-TCAGTATGAGCCTGCTGGTTTGG-3’ and reverse 5’-CCGGGTCTGATGATGTATGCTACC-3’; ‘GGTCTGATGATGTATGCT–GGGCAGTAATCTCCTTCTGCATCC-3’ and reverse 5’-TTCCTGGGTATGGAATCCTGTGG-3’.

### Western blot analysis

In Western Blot analysis, whole cell lysate was prepared from primary culture of macrophages treated with indicated stimuli in the Results or Figure Legends by RIPA buffer (Sigma Aldrich, St. Louis, MO) supplemented with proteinase and phosphatase inhibitors (Roche, Indianapolis, IN). After Sodium Dodecyl Sulfate- Polyacrylamide gel Electrophoresis (SDS-PAGE), separated proteins were transferred to a PVDF membrane (Immobilon-P, Merck) and blocked with an ECL plus blocking agent (GE healthcare, Buckinghamshire, UK). Blotted membranes were, then, incubated with primary antibodies followed by incubation with anti-IgG antibody conjugated with horseradish peroxidase (GE healthcare). Finally, the signal was detected by the chemiluminescent reagent (ECL Prime Western Blotting Detection System, GE healthcare). α-Tubulin was served as an internal control.

Primary antibodies used in this study are as follows: rabbit monoclonal anti-p65 antibody (Cell Signaling Technology, Danvers, MA), rabbit monoclonal anti-phospho NF-κB p65 (ser536) antibody (Cell Signaling Technology), mouse monoclonal anti-IκBα antibody (Cell Signaling Technology), mouse monoclonal anti-α-tubulin antibody (Sigma Aldrich).

### Phagocytic activity of BMM

BMM prepared as described above were cultured with an iron-containing nanoparticle (ferumoxytol, AMAG Pharmaceuticals, Inc., Waltham, MA) for 24 h. Cells were then fixed and iron engulfed by BMM was detected after Berlin blue staining.

### Human IA specimen and immunohistochemistry

Human IA samples and control arteries (middle meningeal artery or superficial temporal artery) were obtained during neck clipping of IAs with written informed consent. The use of human IAs in research was approved by the local ethical committee at Kyoto University Graduate School of Medicine and also at Japan Red Cross Asahikawa Hospital.

Dissected specimen of IAs and control arterial walls were fixed in formalin solution and embedded in paraffin. Slices with 4 μm thickness were then prepared for immunohistochemical analysis. Immunohistochemistry was done same as described in the previous section after deparaffinization.

Primary antibodies used are: mouse monoclonal anti-human CD3 antibody (#NCL-CD3-PS1, Leica Biosystems, Wetzlar, Germany), mouse monoclonal anti-human CD4 antibody (#NCL-CD4-1F6, Leica Biosystems), mouse monoclonal anti-human CD8 antibody (#M7103, Dako).

### Statistical analysis

Data are shown as the mean ± SEM, and 2 groups were statistically compared using the Mann−Whitney U test. A p value smaller than 0.05 was defined as statistically significant.

## Results

### Infiltration of T cells in human IA lesions

**Fig 1:**
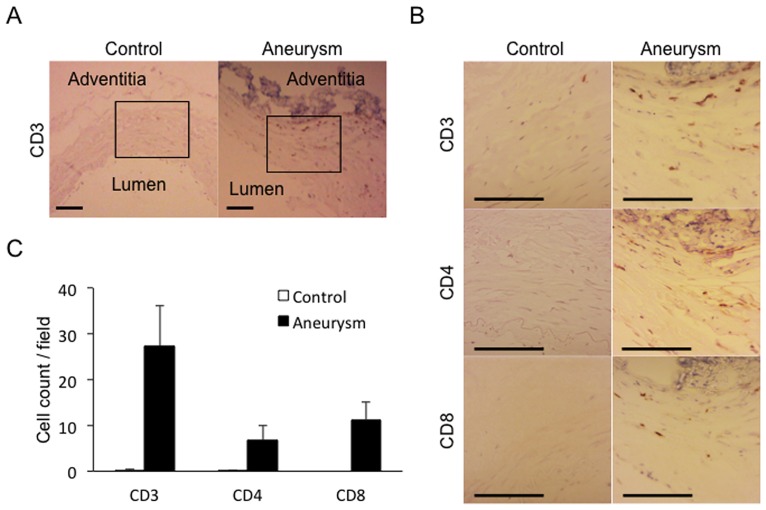
Accumulation of T cells in human unruptured IA walls. (A, B) Accumulation of CD3-, CD4- and CD8-positive T cells in human unruptured IA walls. Adjacent sections were prepared from branches of carotid artery (Control) or unruptured IA lesion (Aneurysm) of humans and immunostained for a T cell marker, CD3, or subset specific makers, CD4 and CD8, respectively. Box in (A) indicates the region magnified in the following panels shown in (B). Bar, 100 μm. (C) Number of CD3-, CD4- and CD8-positive T cells in control arterial walls and IA walls. Data represents mean ± SEM (n = 10 in control, n = 15 in aneurysm). *, p<0.05, **, p<0.01 in a Mann−Whitney U test. Noted the significant increase in both CD4- and CD8-positive T cells in IA walls compared with those in control arterial walls.

First, we wished to confirm and reproduce the previously reported findings that T cells are present and accumulated in human IA lesions [12, 13]. We therefore collected 5 human IA lesions and 2 control arterial walls (superficial temporal artery or middle meningeal artery) and immunostained sections from these specimens using an anti-CD3 antibody. In all IA walls examined, signals positive for CD3 were detectable as ones scattered in media and adventitia (27.3 ± 8.9 cells / high-power field (HPF, 56,000 μm^2^), 5 fields / section, n = 5) as contrasted with those in control arterial walls, in which signals were almost absent (0.2 ± 0.1 cells / HPF, 5 fields / section, n = 2) (Fig 1). We next characterized a subset of T cells infiltrated in IA walls and found that CD3-positive T cells present in IA walls included both CD4-positive and CD8-positive T cell subsets and their number was not significantly different (CD4; 6.8 ± 3.2 cells / HPF, CD8; 11.3 ± 3.7 cells / HPF) (Fig 1B and 1C). Noted that number of CD3-, CD4- and CD8-positive T cells was all significantly increased in IA lesions compared with control arterial walls (CD3; p<0.001, CD4; p = 0.047, CD8; p<0.001), suggesting the role of T cell function in IA formation and progression.

### Involvement of T cell function in IA formation and progression examined by using T cell-deficient F344/NJcl-rnu/rnu rats

**Fig 2:**
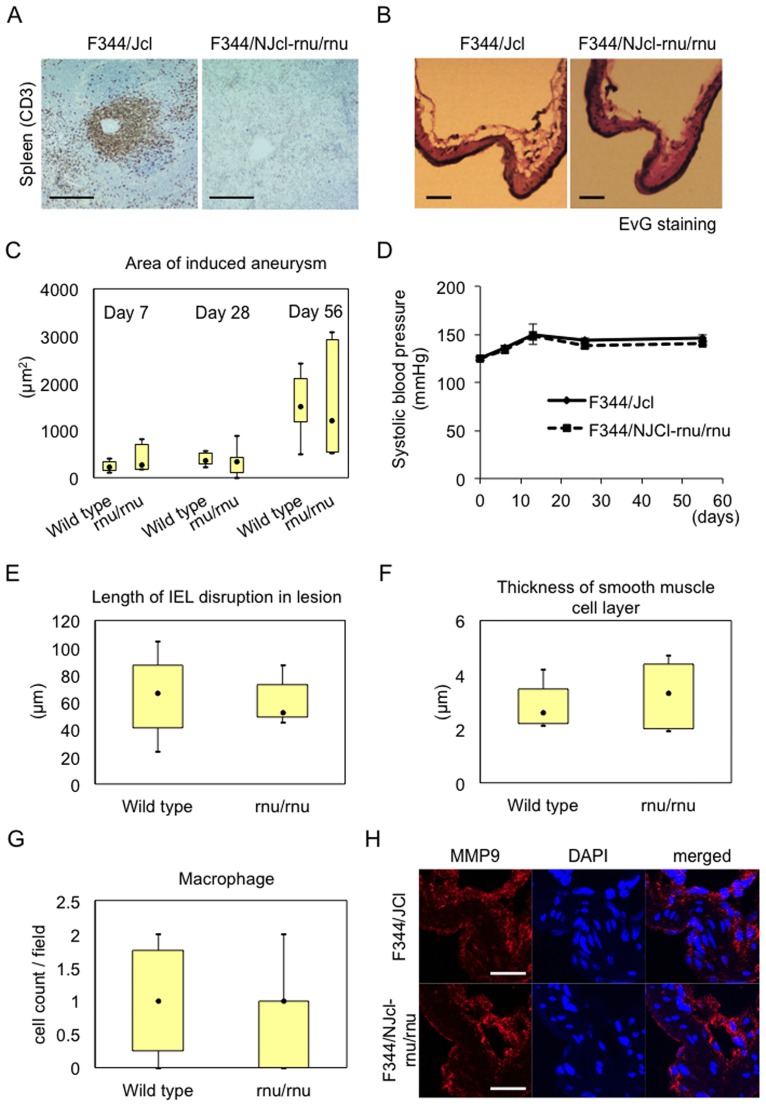
Effect of T cell deficiency on IA progression. (A) Deficiency in T cells in a F344/NJcl-rnu/rnu rat. Sections prepared from spleen from a T cell-deficient F344/NJcl-rnu/rnu rat and a wild type F344/Jcl were immunostained for a T cell marker, CD3. Images from immunostaining for CD3 (green), a nuclear staining DAPI (blue) and merged images are shown. Bar, 20 μm. Noted the deficiency in CD3-positive T cells in a F344/NJcl-rnu/rnu rat. (B-G) Independence of IA progression, systemic blood pressure, degenerative changes and macrophage infiltration in lesions from deficiency in T cells. F344/NJcl-rnu/rnu rats and F344/Jcl rats were subjected to IA induction and, at indicated days (7^th^, 28^th^ and 56^th^) after the induction, slices from IA specimen induced at an anterior cerebral-olfactory artery (ACA-OA) bifurcation were prepared for further analyses. Systemic blood pressure was evaluated before sacrifice without any anesthesia and mean from 3 independent measurements was calculated (D). Area of induced IAs (F344/Jcl: day 7 (n = 8), 28 (n = 8), or day 56 (n = 8); F344/NJcl-rnu/rnu: day 7 (n = 7), 28 (n = 7), or day 56 (n = 7)) (C) and distance between disrupted internal elastic lamina (IEL) in lesions (F344/Jcl: n = 8, F344/NJcl-rnu/rnu: n = 7, day 56) (E) were evaluated after Elastica van Gieson (EvG) staining (Representative images are shown in B). Degenerative change in IA walls was assessed by thickness of medial smooth muscle cell layer after immunostaining for α-smooth muscle actin, a smooth muscle cell marker (F344/Jcl: n = 8, F344/NJcl-rnu/rnu: n = 7, day 56) (F). Number of infiltrated macrophages in IA lesions was evaluated after immunostaining for a macrophage marker CD68 (F344/Jcl: n = 8, F344/NJcl-rnu/rnu: n = 7, day 56, cell count per 12,100 μm^2^). Data represents mean ± SEM. (H) MMP9 expression in IA lesion from both strains. Slices of induced IA lesions from both strains were prepared as above at day 7 after the induction and subjected to immunostaining for MMP9. Images from immunostaining for MMP9 (red), a nuclear staining DAPI (blue) and merged images are shown. Bar, 20 μm in (B), 10 μm in (H). Noted that there are not any differences identified in either evaluation between T cell-deficient- and wild type rats.

Next, to examine whether T cells are involved in the pathogenesis of IA formation and progression and, if so, how, we used a rat strain deficient in T cells due to a mutation in *Whn* gene, a F344/NJcl-rnu/rnu rat [16], and a F344/Jcl rat as a wild type control. First, we confirmed that CD3-positive T cell was indeed absent in F344/NJcl-rnu/rnu rat in immunohistochemistry using the spleen from this rat strain as a specimen and that from F344/Jcl rat as a wild type control (Fig 2A). We then subjected these rat strains to IA model and evaluated whether T cell deficiency affected IA formation and progression. IA defined as a lesion with disrupted internal elastic lamina was induced in all rats examined independent of genotype. Both strains of rats, a F344/Jcl and a F344/NJcl-rnu/rnu rat, showed similar IA progression after the aneurysm induction examined by area of induced IAs (F344/Jcl rat; 232.6 ± 102.3, 385.9 ± 40.1, 1551.4 ± 200.1 μm^2^ for day 7 (n = 8), 28 (n = 8), or day 56 (n = 8), respectively, F344/NJcl-rnu/rnu rat; 379.9 ± 254.2, 395.5 ± 140.8, 1572.2 ± 421.0 μm^2^ for day 7 (n = 7), 28 (n = 7), or day 56 (n = 7), respectively) (Fig 2B and 2C). Since IA formation and progression in rodent IA model is influenced by systemic blood pressure [18, 19], systemic blood pressure at each time point was evaluated and there was no significant difference (Fig 2D). Consistently, deficiency of T cells in rats did not affect degenerative changes in IA walls, disruption of internal elastic lamina examined by Elastica van Gieson staining (F344/Jcl rat; 65.5 ± 8.9 μm, n = 8, F344/NJcl-rnu/rnu rat; 59.2 ± 5.8 μm, n = 7) and thinning of media evaluated by immunostaining for α-SMA, at 56^th^ day after the induction (F344/Jcl rat; 2.9 ± 0.3 μm, n = 8, F344/NJcl-rnu/rnu rat; 3.3 ± 0.4 μm, n = 7) (Fig 2E and 2F). Macrophage is a major inflammatory cell in IA lesions and plays a crucial role in formation and progression of the disease [7–10]. Thereby, we examine infiltration of CD68-positive macrophages in IA lesions in immunohistochemistry. As expected from the similar progression of IAs and similar degeneration of arterial walls in IAs induced in F344/NJcl-rnu/rnu rats (Fig 2B–2F), number of infiltrated macrophages in lesions was not affected by deficiency in T cells (F344/Jcl rat; 1.0 ± 0.3 cells / HPF, n = 8, F344/NJcl-rnu/rnu rat; 0.9 ± 0.2 cells / HPF, n = 7) (Fig 2G). Consistently, expression of facilitating factor of IA progression secreted from macrophages, MMP9 [7, 14], was not changed in IA lesions from F344/NJcl-rnu/rnu rats compared with those from control F344/Jcl rats (Fig 2H).

**Fig 3:**
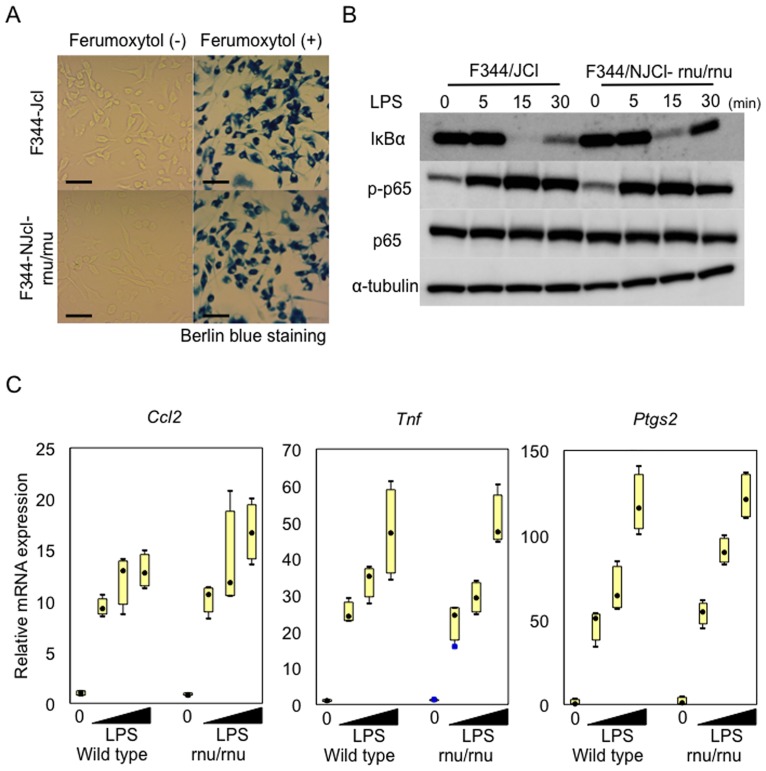
Proper macrophage activation in a T cell-deficient F344/NJcl-rnu/rnu rat. Bone marrow macrophages (BMMs) were prepared from a T cell-deficient F344/NJcl-rnu/rnu rat and a wild type F344/Jcl rat as described in Materials and Methods in detail. Phagocytic activity (A) and response to inflammatory stimulation with LPS (B, C) of BMMs prepared were then evaluated. To examine phagocytic activity, BMMs from both strains were treated with iron-containing nanoparticles, Ferumoxytol, for 24 h and engulfed particles were detected by Berlin blue staining (A). Bar, 20 μm. Response of BMMs from both strains to LPS was assessed by Western blot analysis and RT-PCR analyses targeting NF-κB-regulated pro-inflammatory genes. For Western blotting, BMMs were stimulated with LPS (1 μg/ml) for indicated time period and total cell lysate prepared from these stimulated cells was subjected to Western blotting for IκBα, phosphorylated form of p65 subunit (s536, p-p65) and p65 subunit to evaluate NF-κB activation, using Western blotting for α-tubulin as an internal control. For RT-PCR analyses, total RNA was purified from stimulated cells (LPS 0.1, 1, 10 μg/ml, 60 min) and subjected to RT-PCR analyses for MCP-1 (*Ccl2*), TNF-α (*Tnf*) and COX-2 (*Ptgs2*) after reverse transcription. Data represents mean ± SEM (n = 4). Noted that BMMs from a T cell-deficient rat shows a similar phagocytic activity and properly responses to inflammatory stimulation as in a wild type BMMs.

As macrophage plays a crucial role in IA formation and progression [7–10], we wished to confirm that deficiency in T cells in F344/NJcl-rnu/rnu rats was indeed dispensable for function/activation of macrophages. To address this issue, we prepared BMMs from a F344/NJcl-rnu/rnu rat and a control F344/Jcl rat and examined whether there are any differences in activation of macrophages between those prepared from these two rat stains. As a result, BMMs from these two strains both showed a similar phagocytic activity examined by engulfment of iron-containing nanoparticles, Ferumoxytol, followed by detection with Berlin Blue staining (Fig 3A). Furthermore, BMMs from a F344/NJcl-rnu/rnu rat responded to stimulation with LPS (1 μg/ml) to activate NF-κB, as evidence by stimulation-dependent degradation of IκBα and increase in phosphorylation of NF-κB p65 subunit, (Fig 3B) and to induce genes of pro-inflammatory factors related with the pathogenesis of IAs, MCP-1 (*Ccl2*) [7, 8], TNF-α (*Tnf*) [20–22], and COX-2 (*Ptgs2*) [23] (Fig 3C) in a similar manner with BMMs from a F344/Jcl rat.

### Effect of pharmacological inhibition of T cell function on IA formation and progression

**Fig 4:**
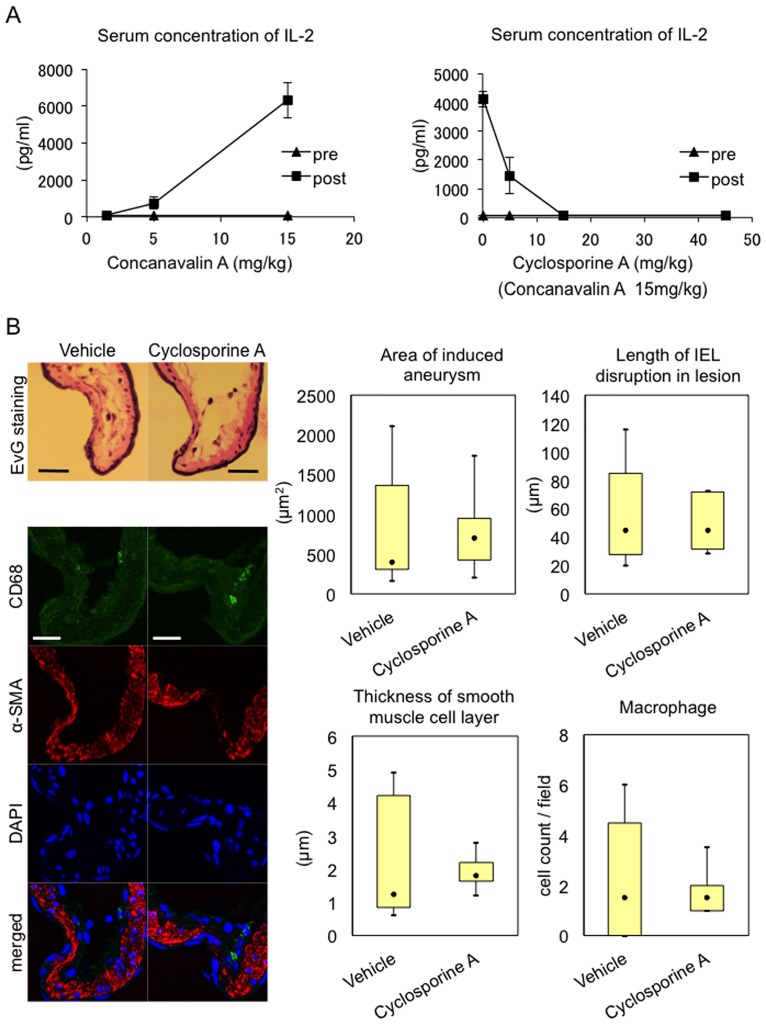
Effect of a pharmacological inhibition of a T cell function on IA progression. (A) Dose-dependent suppression of Concanavalin A-induced IL-2 production by Cyclosporine A *in vivo*. F344/Jcl rats were treated with each dose of Concanavalin A (n = 3) or Cyclosporine A combined with 15 mg/kg Concanavaline A (n = 3). Before administration (pre) or after 3 h (post), serum samples were prepared from rats and IL-2 concentration in these samples was examined by ELISA. Noted that 15 mg/kg Cyclosporine A almost completely suppressed Concanavalin A-induced IL-2 production. (B) Failure of suppression of IA progression, degenerative changes and infiltration of macrophages in lesions by treatment with Cyclosporine A. F344/Jcl rats were subjected to IA induction and, at 14^th^ day after the induction, slices from IA specimen induced at ACA-OA bifurcation were prepared for further analyses. Administration of Cyclosporine A was started 1 day before the induction and lasted for a whole experimental period (15 mg/kg, once a day). Systemic blood pressure was evaluated before sacrifice without any anesthesia. Area of induced IAs (Vehicle; n = 8, Cyclosporine A; n = 8) and distance between disrupted internal elastic lamina (IEL) in lesions were evaluated after Elastica van Gieson (EvG) staining (Representative images are shown in upper left panels). Degenerative change in IA walls was assessed by thickness of medial smooth muscle cell layer after immunostaining for α-smooth muscle actin. Number of infiltrated macrophages in IA lesions was evaluated after immunostaining for a macrophage marker CD68 (per 12,100 μm^2^). Data represents mean ± SEM. Representative images of immunostaining for CD68 (green) and α-smooth muscle actin (α-SMA, red) and of nuclear staining DAPI (blue) are shown in lower left panels. Bar, 20 μm.

To further corroborate our assumption that T cell function is dispensable for the pathogenesis of IA, we administered an immunosuppressant, Cyclosporine A, to rat IA model and examined effect of pharmacological suppression of T cell function on IA formation and progression. In this experiment, we adopted 15 mg/kg as a dose of Cyclosporine A used from the preliminary study in which administration of this compound suppressed Concanavalin A (15 mg/kg)—induced increase in plasma concentration of IL-2 at a dose-dependent manner and at this or more dose of Cyclosporine A such increase was completely ameliorated (Fig 4A). Cyclosporine A (15 mg/kg) administered to rat model of IAs neither suppressed IA progression nor degenerative changes of arterial walls; thinning of media and disruption of internal elastic lamina (Fig 4B). Furthermore, inflammation in lesions characterized by infiltration of macrophages was not affected by Cyclosporine A treatment (Fig 4B). These results combined with findings obtained from a T cell-deficient F344/NJcl-rnu/rnu rat strain suggest that T cell is dispensable for the pathogenesis of IA progression.

## Discussion

The pathological examination of human IA walls has revealed the presence of inflammatory infiltrates, most typically macrophages and T cells [12–14], in lesions proposing the involvement of inflammation evoked by these infiltrating cells in the pathogenesis of IA formation and progression. Indeed, recent experimental studies have clarified the crucial contribution of macrophages recruited by their chemoattractant MCP-1 to these processes through mediating and exacerbating inflammation [7, 8, 10, 11]. However, the central question whether T cells actively participate in the pathogenesis of IAs and, if so, how they contribute to the pathogenesis remains unsolved for about 20 years after the presence of T cells in IA lesions was histologically detected and reported [12, 13]. In the present study, we addressed this remaining but important question by subjecting T cell-deficient rats to IA models or pharmacological inhibition of T cell function and demonstrated that T cell function is dispensable for IA formation and progression. As T cells have distinct subsets and each subset sometimes oppositely functions, a subset-specific depletion of T cells may reveal some contribution of a certain subset of T cells to the pathogenesis of IAs. Recently published report demonstrating the progressive bilateral fusiform intracranial aneurysms in a child with human immunodeficiency virus/acquired immunodeficiency syndrome, in which CD4-positive T cell subset is affected, indicates such a possibility [24]. Recently, Sawyer et al. also reported the similar observation with us in mouse IA model [25]. In their report, they subjected mice deficient in T cells (*Rag1-/-*) to IA models, in which IAs were induced by surgical manipulations similar with our models, and found that T cell deficiency did not affect IA formation[25] as in our results using a T cell-deficient rat model, supporting our notion that macrophages not T cells regulate the pathogenesis of IAs. Thus, findings of the present study have again highlighted the importance of macrophages as the major type of cells regulating inflammatory responses leading to IA formation and progression. Atherosclerosis is presumably one of the most important and common vascular diseases in society. Both IAs and atherosclerosis share the common pathogenesis as a chronic inflammatory disease in arterial walls [11, 26]. The contribution of T cells to disease development and progression, however, may be completely different. In atherosclerosis, T cells, especially a CD4-positive T cell subset, actively participate in its pathogenesis; i.e. Th1 cells play a proatherogenic role but regulatory T cells behave atheroprotective[27–30], suggesting presence of completely different regulation of inflammatory responses between these two vascular diseases. Now, IA is defined as a macrophage-mediated chronic inflammatory disease of intracranial arteries. This concept is pivotal not only for our understanding of the pathogenesis of IAs but also for developing a novel diagnostic method to monitor inflammatory status of IA walls or a new drug therapy.

The major limitation of the present study is that we cannot assess the contribution of T cell-mediated acquired immunity to rupture of IAs because the rupture rate of IAs induced in our current model is not high enough to statistically analyze the influence of intrinsic or confounding factors. To address such an imperative issue, a novel animal model, in which IAs harbor the pathological similarity with human ones and spontaneously rupture at high rate, should be established. Although careful interpretation is necessary regarding the clinical relevance of their mouse IA model, Sawyer et al. demonstrated the suppression of rupture of IAs in mice deficient in T cells [25], suggesting the involvement of T cells in mechanisms underlying rupture of IAs. If so, there may be different pathogenesis between formation/progression and rupture of IAs. In this point of view, contribution of each T cell subset, such as a CD4-positive T cell, a CD8-positive T cell and a regulatory T cell, to rupture of IAs should be carefully examined. Nonetheless, given results from recent clinical studies that anti-inflammatory drugs such as statins and NSAIDs effectively reduce incidence of subarachnoid hemorrhage [31, 32], chronic inflammation in arterial walls may regulates the pathogenesis of IAs from initiation to rupture even if the actively participating type of cells are different.

## Supporting information

S1 Fig Law data of T cells in human IA. (XLSX)

S2 Fig Law data of IA formation examined by using T cell-deficient rats. (XLSX)

S3 Fig Law data of blood pressure of T cell-deficient rats. (XLSX)

S4 Fig Law data of PCR of bone marrow macrophages. (XLSX)

S5 Fig Law data of western blotting of bone marrow macrophages. (PPTX)

S6 Fig Law data of ELISA of Concanavalin A induced increase in plasma concentration of IL-2. (XLS)

S7 Fig Law data of ELISA of suppressed IL-2 by Cyclosporin A. (XLSX)

S8 Fig Law data of IA formation examined by pharmacological T cell suppression. (XLSX)

## References

[1] van Gijn J, Kerr RS, Rinkel GJ. Subarachnoid haemorrhage. Lancet. 2007;369(9558):306–318. Epub 2007/01/30. 10.1016/S0140-6736(07)60153-6 17258671

[2] Wermer MJ, Kool H, Albrecht KW, Rinkel GJ. Subarachnoid hemorrhage treated with clipping: long-term effects on employment, relationships, personality, and mood. Neurosurgery. 2007;60(1):91–97; discussion 97–98. Epub 2007/01/18. 10.1227/01.NEU.0000249215.19591.86 17228256

[3] Iwamoto H, Kiyohara Y, Fujishima M, Kato I, Nakayama K, Sueishi K, et al Prevalence of intracranial saccular aneurysms in a Japanese community based on a consecutive autopsy series during a 30-year observation period. The Hisayama study. Stroke. 1999;30(7):1390–1395. Epub 1999/07/02. 10390312 10.1161/01.str.30.7.1390

[4] Sonobe M, Yamazaki T, Yonekura M, Kikuchi H. Small unruptured intracranial aneurysm verification study: SUAVe study, Japan. Stroke. 2010;41(9):1969–1977. Epub 2010/07/31. 10.1161/STROKEAHA.110.585059 20671254

[5] Morita A, Kirino T, Hashi K, Aoki N, Fukuhara S, Hashimoto N, et al The natural course of unruptured cerebral aneurysms in a Japanese cohort. N Engl J Med. 2012;366(26):2474–2482. Epub 2012/06/29. 10.1056/NEJMoa1113260 22738097

[6] Rinkel GJ, Djibuti M, Algra A, van Gijn J. Prevalence and risk of rupture of intracranial aneurysms: a systematic review. Stroke. 1998;29(1):251–256. Epub 1998/01/28. 9445359 10.1161/01.str.29.1.251

[7] Aoki T, Kataoka H, Ishibashi R, Nozaki K, Egashira K, Hashimoto N. Impact of monocyte chemoattractant protein-1 deficiency on cerebral aneurysm formation. Stroke. 2009;40(3):942–951. Epub 2009/01/24. 10.1161/STROKEAHA.108.532556 19164781

[8] Kanematsu Y, Kanematsu M, Kurihara C, Tada Y, Tsou TL, van Rooijen N, et al Critical roles of macrophages in the formation of intracranial aneurysm. Stroke. 2011;42(1):173–178. Epub 2010/11/26. 10.1161/STROKEAHA.110.590976 21106959 PMC3021554

[9] Frosen J, Tulamo R, Paetau A, Laaksamo E, Korja M, Laakso A, et al Saccular intracranial aneurysm: pathology and mechanisms. Acta Neuropathol. 2012;123(6):773–786. Epub 2012/01/18. 10.1007/s00401-011-0939-3 22249619

[10] Fukuda M, Aoki T. Molecular basis for intracranial aneurysm formation. Acta Neurochir Suppl. 2015;120:13–15. Epub 2014/11/05. 10.1007/978-3-319-04981-6_2 25366592

[11] Aoki T, Narumiya S. Prostaglandins and chronic inflammation. Trends Pharmacol Sci. 2012;33(6):304–311. Epub 2012/04/03. 10.1016/j.tips.2012.02.004 22464140

[12] Chyatte D, Bruno G, Desai S, Todor DR. Inflammation and intracranial aneurysms. Neurosurgery. 1999;45(5):1137–1146; discussion 1146–1137. Epub 1999/11/05. 10549930 10.1097/00006123-199911000-00024

[13] Frosen J, Piippo A, Paetau A, Kangasniemi M, Niemela M, Hernesniemi J, et al Growth factor receptor expression and remodeling of saccular cerebral artery aneurysm walls: implications for biological therapy preventing rupture. Neurosurgery. 2006;58(3):534–541; discussion 534–541. Epub 2006/03/11. 10.1227/01.NEU.0000197332.55054.C8 16528195

[14] Aoki T, Kataoka H, Morimoto M, Nozaki K, Hashimoto N. Macrophage-derived matrix metalloproteinase-2 and -9 promote the progression of cerebral aneurysms in rats. Stroke. 2007;38(1):162–169. Epub 2006/11/24. 10.1161/01.STR.0000252129.18605.c8 17122420

[15] Frosen J, Piippo A, Paetau A, Kangasniemi M, Niemela M, Hernesniemi J, et al Remodeling of saccular cerebral artery aneurysm wall is associated with rupture: histological analysis of 24 unruptured and 42 ruptured cases. Stroke. 2004;35(10):2287–2293. Epub 2004/08/24. 10.1161/01.STR.0000140636.30204.da 15322297

[16] Nehls M, Pfeifer D, Schorpp M, Hedrich H, Boehm T. New member of the winged-helix protein family disrupted in mouse and rat nude mutations. Nature. 1994;372(6501):103–107. Epub 1994/11/03. 10.1038/372103a0 7969402

[17] Aoki T, Nishimura M. The development and the use of experimental animal models to study the underlying mechanisms of CA formation. J Biomed Biotechnol. 2011;2011:535921 Epub 2011/01/22. 10.1155/2011/535921 21253583 PMC3018658

[18] Nagata I, Handa H, Hashimoto N, Hazama F. Experimentally induced cerebral aneurysms in rats: Part VI. Hypertension. Surg Neurol. 1980;14(6):477–479. Epub 1980/12/01. 6111849

[19] Hashimoto N, Handa H, Hazama F. Experimentally induced cerebral aneurysms in rats. Surg Neurol. 1978;10(1):3–8. Epub 1978/07/01. 684603

[20] Aoki T, Fukuda M, Nishimura M, Nozaki K, Narumiya S. Critical role of TNF-alpha-TNFR1 signaling in intracranial aneurysm formation. Acta Neuropathol Commun. 2014;2:34 Epub 2014/04/02. 10.1186/2051-5960-2-34 24685329 PMC3974421

[21] Yokoi T, Isono T, Saitoh M, Yoshimura Y, Nozaki K. Suppression of cerebral aneurysm formation in rats by a tumor necrosis factor-alpha inhibitor. J Neurosurg. 2014;120(5):1193–1200. Epub 2014/03/19. 10.3171/2014.1.JNS13818 24628611

[22] Starke RM, Chalouhi N, Jabbour PM, Tjoumakaris SI, Gonzalez LF, Rosenwasser RH, et al Critical role of TNF-alpha in cerebral aneurysm formation and progression to rupture. J Neuroinflammation. 2014;11:77 Epub 2014/04/18. 10.1186/1742-2094-11-77 24739142 PMC4022343

[23] Aoki T, Nishimura M, Matsuoka T, Yamamoto K, Furuyashiki T, Kataoka H, et al PGE(2) -EP(2) signalling in endothelium is activated by haemodynamic stress and induces cerebral aneurysm through an amplifying loop via NF-kappaB. Br J Pharmacol. 2011;163(6):1237–1249. Epub 2011/03/24. 10.1111/j.1476-5381.2011.01358.x 21426319 PMC3144537

[24] Piantino JA, Goldenberg FD, Pytel P, Wagner-Weiner L, Ansari SA. Progressive intracranial fusiform aneurysms and T-cell immunodeficiency. Pediatr Neurol. 2013;48(2):130–134. Epub 2013/01/23. 10.1016/j.pediatrneurol.2012.10.004 23337006

[25] Sawyer DM, Pace LA, Pascale CL, Kutchin AC, O'Neill BE, Starke RM, et al Lymphocytes influence intracranial aneurysm formation and rupture: role of extracellular matrix remodeling and phenotypic modulation of vascular smooth muscle cells. J Neuroinflammation. 2016;13(1):185 Epub 2016/07/16. 10.1186/s12974-016-0654-z 27416931 PMC4946206

[26] Rader DJ, Daugherty A. Translating molecular discoveries into new therapies for atherosclerosis. Nature. 2008;451(7181):904–913. Epub 2008/02/22. 10.1038/nature06796 18288179

[27] Witztum JL, Lichtman AH. The influence of innate and adaptive immune responses on atherosclerosis. Annu Rev Pathol. 2014;9:73–102. Epub 2013/08/14. 10.1146/annurev-pathol-020712-163936 23937439 PMC3988528

[28] Foks AC, Lichtman AH, Kuiper J. Treating atherosclerosis with regulatory T cells. Arterioscler Thromb Vasc Biol. 2015;35(2):280–287. Epub 2014/11/22. 10.1161/ATVBAHA.114.303568 25414253 PMC4715365

[29] Hedrick CC. Lymphocytes in atherosclerosis. Arterioscler Thromb Vasc Biol. 2015;35(2):253–257. Epub 2015/01/23. 10.1161/ATVBAHA.114.305144 25609772 PMC4327776

[30] Meng X, Yang J, Dong M, Zhang K, Tu E, Gao Q, et al Regulatory T cells in cardiovascular diseases. Nat Rev Cardiol. 2016;13(3):167–179. Epub 2015/11/04. 10.1038/nrcardio.2015.169 26525543 PMC11849084

[31] Hasan DM, Mahaney KB, Brown RD Jr., Meissner I, Piepgras DG, Huston J, et al Aspirin as a promising agent for decreasing incidence of cerebral aneurysm rupture. Stroke. 2011;42(11):3156–3162. Epub 2011/10/08. 10.1161/STROKEAHA.111.619411 21980208 PMC3432499

[32] Yoshimura Y, Murakami Y, Saitoh M, Yokoi T, Aoki T, Miura K, et al Statin use and risk of cerebral aneurysm rupture: a hospital-based case-control study in Japan. J Stroke Cerebrovasc Dis. 2014;23(2):343–348. Epub 2013/05/24. 10.1016/j.jstrokecerebrovasdis.2013.04.022 23697760

