# Supplementary figures and images for: T cell function is dispensable for intracranial aneurysm formation and progression
Source: PLoS One. 2017 Apr 24;12(4):e0175421. doi: 10.1371/journal.pone.0175421 (PMC5402951; doi:10.1371/journal.pone.0175421)

## Slide 1
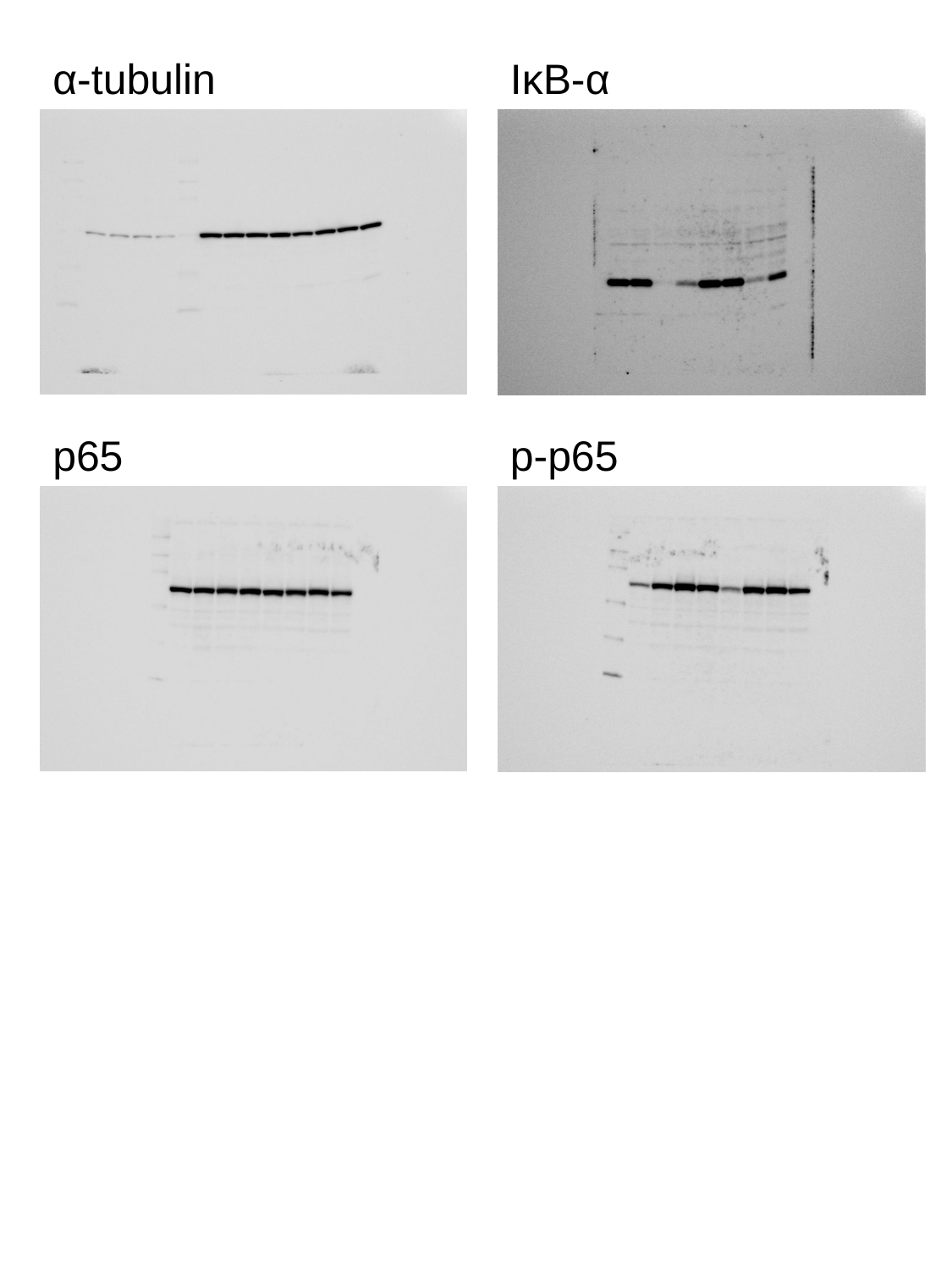

IκB-α
α-tubulin
p65
p-p65

Supplement: S5 Fig — (PPTX) [file pone.0175421.s005.pptx]
